# Primary cerebellar endodermal sinus tumor: A case report

**DOI:** 10.3892/ol.2014.2340

**Published:** 2014-07-10

**Authors:** HONGMEI KUANG, CHUN ZHANG, HONGHAN GONG, LINGHONG GUO, CHEN YU, XIANJUN ZENG

**Affiliations:** Department of Radiology, The First Affiliated Hospital of Nanchang University, Nanchang, Jiangxi 330006, P.R. China

**Keywords:** endodermal sinus tumor, cerebellar hemisphere, extragonadal

## Abstract

Endodermal sinus tumors are rare malignant germ cell tumors that usually originate from the gonads and are rarely observed extragonadally. Pure primary endodermal sinus tumors of the cerebellar hemisphere are extremely rare and patients diagnosed with the disease often have a poor prognosis. The symptoms of YSTs are unspecific and associated with the location of tumors. Intracranial YSTs (such as cerebellar hemispheres) always present with symptoms including headache and poor vision. The present study reports the case of a three-year-old male who presented to The First Affiliated Hospital of Nanchang University (Nanchang, China) with a headache that had persisted for one month, and then worsened for the last 10 days. This was accompanied by vomiting and gait disturbance. An abnormal signal mass was identified in the left cerebellar hemisphere on brain magnetic resonance imaging. The case initially presented as a medulloblastoma and the patient was followed up for six months. The final pathology report revealed an endodermal sinus tumor, also known as a yolk sac tumor. Six months following resection of the left cerebellar tumor, the patient succumbed to recurrence of the disease, due to acute vomiting and severe headache.

## Introduction

At present, no studies have analyzed the total incidence of yolk sac tumors (YSTs), however, it has been reported that YSTs most commonly occur in the pediatric testis (1). Pediatric germ cell tumors account for 60–75% of pediatric testicular tumors, mostly as YSTs. The occurrence of the tumor in the cerebellar hemisphere is extremely rare and few cases have been reported in the literature (2). Endodermal sinus tumors, also known as YSTs, belong to an inferior class of germ cell tumors (GCTs) with a poor prognosis. The optimal treatment is the surgical resection of the tumor, followed by adjuvant chemotherapy (including bleomycin, etoposide and cisplatin) (3), however, the results are poor. Approximately 80–90% of YSTs arise in the reproductive organs, but may also occur in the extragonadal regions (1,4–12). There have been several previously reported intracranial cases, the majority of which were observed in the pineal region. However, pure primary endodermal sinus tumors that occur in the cerebellar hemisphere are extremely rare (1). The current study presents the case of a three-year-old male with a cerebellar YST, which initially presented as a medulloblastoma. Follow-up was continued for six months. Patient provided written informed consent.

## Case report

A three-year-old male presented to The First Affiliated Hospital of Nanchang University (Nanchang, China) with a headache that had persisted for one month, and then worsened for the last 10 days. This was accompanied by vomiting and gait disturbance. The remainder of the patient’s physical examination and medical, family and surgical histories were unremarkable. At the time of presentation, routine laboratory tests, including a routine blood examination and coagulation indices, were within the normal ranges. Serum tumor markers, including β-human chorionic gonadotropin and α-fetoprotein (AFP), were not measured, as a diagnosis of GCT was not suspected at this stage.

**Figure 1:**
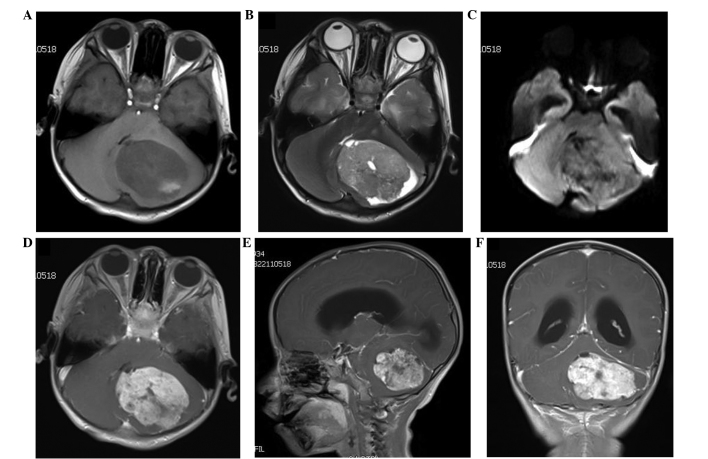
(A-D) Transaxial, (E) sagittal and (F) coronal magnetic resonance imaging prior to surgery, showing a large mass (40-mm maximum diameter). (A) On T1WI, the mass showed a relatively homogeneous uniform signal intensity, with patchy areas of high T1 signal and slight edema around the lesion. (B and C) The fourth ventricle was pushed to the right side and had become narrowed. The tumor exhibited a slightly increased signal intensity on T2- and diffusion-WI. (D–F) T1WI with gadolinium showed a well-demarcated, enhanced mass. WI, weighted imaging.

For further investigation, the patient was referred to the Department of Radiology for brain magnetic resonance imaging (MRI). The imaging revealed an abnormal signal mass in the left cerebellar hemisphere (Fig. 1), but no tumorous lesions were identified at other sites. The MRI clearly revealed the tumors, which showed relatively homogeneous uniform signal intensity on T1-weighted imaging, with patchy areas of a high T1 signal. A slightly increased signal intensity was observed on the T2- and diffusion-weighted images, while the enhanced scan with gadolinium suggested inhomogeneous enhancement. Mild peritumoral edema was also observed around the tumor, and the fourth ventricle was pushed to the right side and had become narrowed. Due to these results, medulloblastoma was initially diagnosed.

**Figure 2:**
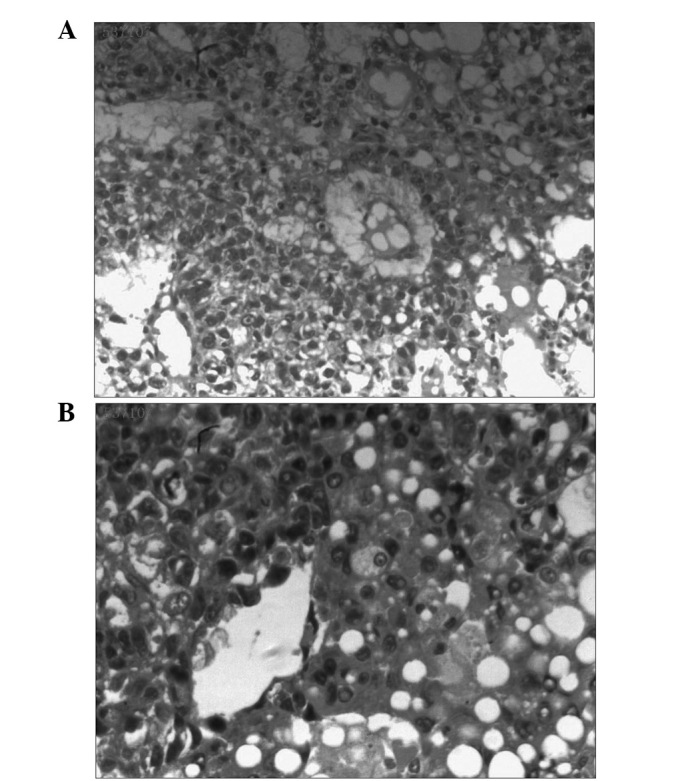
Hematoxylin and eosin staining of (A) diffuse malignant neoplastic cells growing around the blood vessels or cavity, with typical Schiller-Duval bodies (magnification, ×200) and (B) eosinophilic bodies partly existing in the cytoplasm and partly in the extracellular matrix (magnification, ×400).

**Figure 3:**
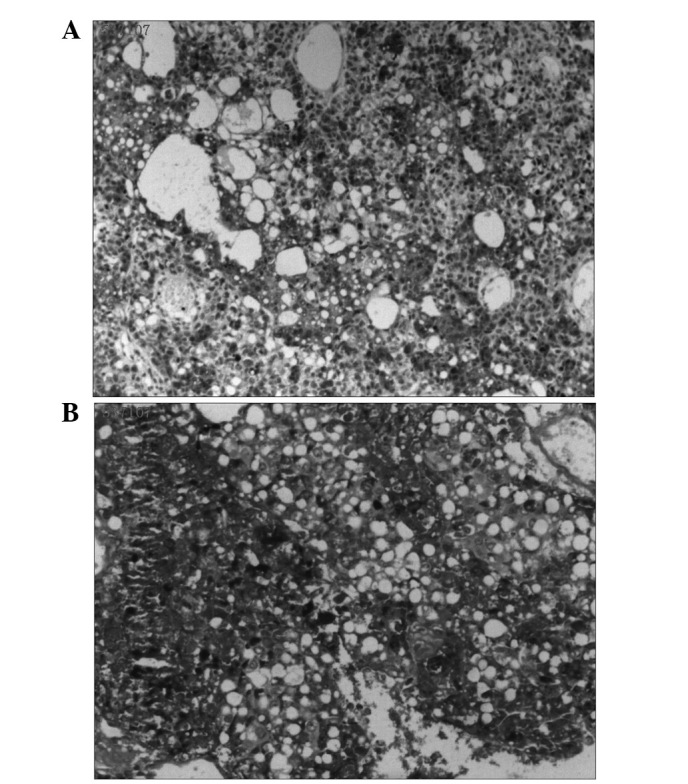
(A) Immunohistochemical staining revealing that the majority of the tumor cells were strongly positive for glypican-3 (magnification, ×100). (B) Periodic acid-Schiff staining showing immunopositivity for eosinophilic bodies (magnification, ×200).

A resection of the left cerebellar tumor was performed. The intraoperative findings revealed a well-defined 4.0×3.0×2.5-cm tumor, with a red and white appearance, an inconsistently soft texture and a rich blood supply. The resected tumor was a solid mass, and the cut surface exhibiteda friable, white-to-tan appearance. The resected specimen was characterized histologically by diffuse, malignant, neoplastic cells proliferating in a microcystic or reticular pattern of growth around the blood vessels or cavity. The neoplastic cells, forming Schiller-Duval bodies, exhibited highly atypical, large nuclei, with evident karyokinesis and eosinophilic bodies that partly existed in the cytoplasm and partly in the extracellular matrix (Fig. 2). Immunohistochemical staining and periodic acid-Schiff (PAS) staining for AFP were strongly positive (Fig. 3). The histological features of the tumorous specimen indicated an endodermal sinus tumor. The cut edge of the left cerebellar hemisphere was not involved, and the vicinity of the tumor was free of tumor cells. Therefore, the diagnosis of an endodermal sinus tumor originating in the left cerebellar hemisphere was determined.

**Figure 4:**
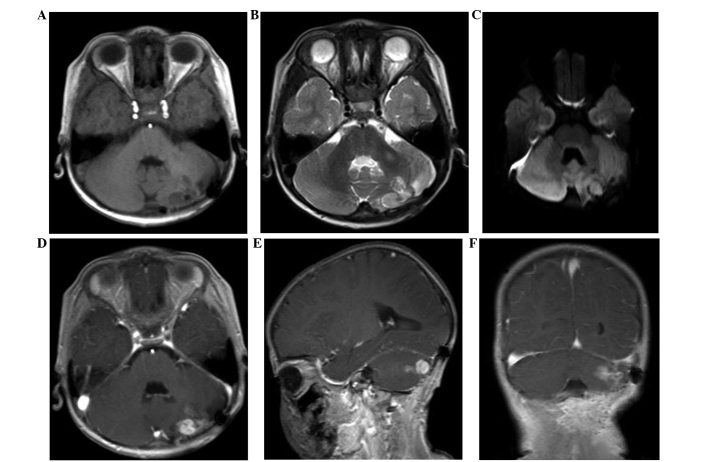
Magnetic resonance imaging of the brain one month after surgery. (A) T1WI, (B) T2WI, (C) diffusion-WI and (D-F) T1WI with gadolinium suggesting that a small relapsing tumor had occurred in the same region. WI, weighted imaging.

**Figure 5:**
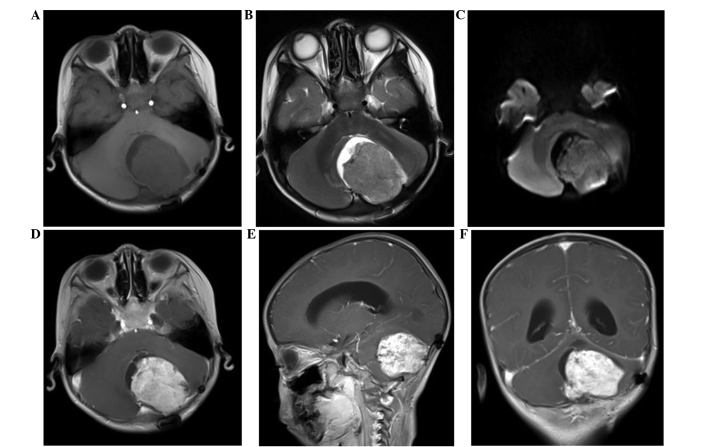
Two months after the surgery, magnetic resonance imaging of the brain on (A) T1WI, (B) T2WI, (C) diffusion-WI and (D-F) T1WI with gadolinium suggesting that the tumor had increased in size following transient expansion. W1, weighted imaging.

Following surgery, the patient’s symptoms were relieved for a while. However, subsequent to one month, MRI of the brain revealed tumor recurrence in the same region (Fig. 4). After another month, the relapsing mass had increased in size (Fig. 5) and the patient’s conditioned had worsened, resulting in the patient succumbing to the disease six months after the diagnosis.

## Discussion

Endodermal sinus tumors are rare malignant GCTs that usually originate from the gonads and are rarely observed extragonadally (4). Several case studies have reported the occurrence of this entity in the vagina, seminal vesicle, omentum, pancreas and stomach, as well as in the sacrococcygeal and intracranial regions (1,5–12). GCTs originating in the intracranial region almost always occur in the pineal gland or suprasellar regions, therefore, a tumor arising in the cerebellar hemisphere is extremely rare (1).

The histogenesis of extragonadal YST remains controversial. There are currently two theories that may explain the occurrence of primary GCT at extragonadal sites; the first is the aberrant differentiation of somatic cells, while the second is the malignant transformation of remnant of germ cells along the pathway of migration (6,7). The latter may be more feasible for explaining the location of the YST in the present case.

The typical morphological structure of the tumor, known as the Schiller-Duval body, is a glomerulus-like structure composed of a monolayer of cubic or columnar neoplastic cells wrapping around the capillaries, thin-walled blood sinus or small venous blood vessels. This forms vessel-centered, sleeve-shaped structures, similar to glomerulus-like structures (6,13).

In the present case, the patient had initially been diagnosed with a medulloblastoma, however, the post-operative pathology confirmed the lesion to be a YST, indicating that caution must be taken when conducting initial examinations in order to avoid misdiagnosis. Initially, the case was consistent with a medulloblastoma with regard to the age distribution and predilection site, and due to its rarity in the cerebellar hemisphere, primary YST was not considered. Furthermore, the clinical and imaging manifestations of YST are not specific. The clinical symptoms are associated with the location of the tumor, whereas the final diagnosis mainly depends on the pathology. Notably, the present patient did not receive the pre-operative tests associated with YST, particularly the test for serum AFP. To the best of our knowledge, the serum AFP levels in YST patients are likely to significantly increase when these types of tumors contain YST elements. It has also been confirmed that an increased AFP level in the serum and cerebrospinal fluid correlates with the presence of a YST tumor (14). In the current case, according to the microscopic pathology, immunohistochemical staining and PAS staining of the tumor, a final diagnosis of YST was determined. Studies have suggested that AFP, glypican-3 and Sal-like protein 4 are sensitive diagnostic markers for YST (6,13). In particular, AFP is useful not only for immunohistochemical staining and pathological diagnosis, but also for assessing the response to treatment and detecting recurrence (1).

Patients diagnosed with YST generally have a poor prognosis (15,16), and in the present case, the infant succumbed to the disease. This may be attributed to the fact that only surgical treatment was administered as opposed to combined chemotherapy, as chemotherapy is known to be an extremely effective treatment, which may improve quality of life and prolong survival time (17).

In conclusion, the current study presents an extremely rare case of primary YST originating in the left cerebellar hemisphere. Extragonadal YST is aggressive and difficult to diagnose, and further investigations are required to define its pre-operative diagnosis and to further optimize the treatment regimen.
